# Sanitary Relations of the Soil*An address delivered before the Association of German Naturalists and Physicians, at Salzburg, September 18, 1881.

**Published:** 1882-05

**Authors:** Max Von Pettenkofer


### Sanitary Relations of the Soil.*

By Dr. Max Von Pettenkofer.

I am well aware that I have chosen no new theme when I assume to speak of our soil and its relations to our health. It is, on the contrary, very old—for Hippocrates wrote two thousand years ago on air, water, and earth in their hygienic relations—but there are old subjects that are always awakening a new interest, and always appear fresh when considered in a new light or from a new side. To these eternally fresh subjects belongs that of the ground on which we stand and live, on which we are born, and in which we are to be buried. Since mankind has comprehended the idea of health, sickness-giving and health-promoting properties have been ascribed to the locality, which has been regarded as consisting of air, water, and earth; but the seat of that which makes sick and makes well has been supposed to be more in the air and water and less in the soil; that is, it has been conceived that a place might have its own air and its own water which we have to use directly in breathing and drinking, while we could be independent of the soil, on which we only tread. The local air could, however, hold the first place in hygienic regimen only as long as it was not known that the average velocity of the atmosphere over the surface of the earth is three metres (or ten feet) in a second, and that, even when we feel that it is perfectly calm, the air is moving at the rate of a

* An address delivered before the Association of German Naturalists and Physicians, at Salzburg, September 18, 1881.

half-metre (or twenty inches) in a second. A real stagnation of the air, even in deep cloves and valleys, or in the narrowest streets, is not to be spoken of; the air is rather to be conceived as undergoing a constant change of place. And if it has properties or contains matters in one place which are not remarked in a neighboring place, they can not originate in the air itself, but must be derived from the locality from which they are communicated to it, and are then carried away in the free atmosphere, to dissappear by dilution and other processes.

The same is the case with the local water. All the water that we drink on the earth falls from the sky, and is everywhere of precisely the same composition. Only when it penetrates the soil is it changed by taking up matter which is derived from the ground through which it flows, a fact that was mentioned by Hippocrates. And the local admixtures disappear from water, partly by dilution, partly by chemical changes, just as they do from the air; only in a lesser degree and more slowly, because water is present in the soil in smaller quantity and moves more sluggishly than air. This purification of the water takes place not only during its continuous retention and movement in porous soils, but also in open river-beds and streams. Brunner and Emmerich have drawn water from the Isar at numerous places between the mountains and the mouth of the river at the Danube, on the same day, and have found it essentially alike everywhere, although the stream receives considerable admixtures from the towns on its banks.

What is there that does not fall into the Elbe in its course from Bohemia down to the sea? Yet filtered Elbe water is considered a pure drinking-water at Hamburg and Altoona,

The river Trent receives, before it reaches Nottingham, the sewer-water of two million people dwelling on its banks, amounting to at least five hundred thousand gallons a day, yet its waters at that city are clear, sweet-smelling, and chemically free from injurious constituents.

At Paris, the collecting sewer of Clichy pours a great stream of blackish water into the gently flowing Seine below the bridge of Asnieres, by which the river is so fouled that neither fish nor plants can live in it; but at Meulan, a few miles below Paris, every trace of impurity has disappeared from the stream.

When the air and water at any place are contaminated, the contamination does not proceed from any combination or decomposition of those two elements, but from qualities of the place, and they soon purify themselves again. An impurity cleaves longest and most tenaciously to the soil, which suffers no change of place, like air and water. While formerly we esteemed the hygienic value of the air first, of the water second, and of the soil third, we should now reverse the order.

The influence of the soil upon the health of those living upon it is brought out very plainly during the prevalence of epidemic diseases. That malarial diseases, like intermittent fevers, originate from the soil, is already accepted; and the more exact studies in recent times of the manner in which cholera, abdominal typhus, yellow fever, and the plague are spread, has convinced many that these diseases, also, which were formerly considered independent of the soil, because their specific germs are communicable and are actually communicated by human intercourse and trade, are still in some way connected with it, although the nature of the connection is yet to be found out. The explanation of the frequent, sharply defined local limitations of cholera and typhoid has been sought first, in influences not of soil but of water and air, to which the germs of disease have been imparted from men; but a clear and impartial examination of the local prevalence of these diseases in circles of greater or lesser extent has now furnished evidence that in many cases air and water can no longer be maintained to be the causes of the localization, but that the sources of the epidemic must be sought in the soil.

In the occurrence of cholera on ships at sea, where any influence of soil would seem to be absolutely out of the question, that influence often makes itself apparent in a striking manner by the fact that only persons who have come from certain places are attacked, while other persons on the ships do not even have a diarrhœa, although they are all the time with the sick, and use the same food and water and air. Ships at sea may be considered as in themselves safe from cholera; usually sickness brought upon them in individual cases dies out; and it is regarded in seafaring practice as an excellent prophylactic measure to go to sea, taking the sick along and breaking up all communication of the men with the infected port or shore. Exceptional cases of epidemics breaking out on ships can not be regarded as arising from contagion from person to person, but always from previous communication of the ship or its crew or passengers with some place infected with the disease.

Not less plainly and frequently is the real influence of the soil shown in inland regions and towns that enjoy immunity from cholera. Permit me to bring forward as a well-known but pregnant example the great manufacturing and commercial city of Lyons, in Southern France, which has constantly maintained with impunity the most active intercourse by sea and land with cities infected with cholera ever since the disease first appeared in Europe. Often as cholera-epidemics have prevailed in Paris and Marseilles, the disease has never yet gained an epidemic footing in Lyons, which lies right between those two cities, notwithstanding many cases have been brought into it from without. Even in 1849, when the city was in revolt and was besieged and occupied by cholera-infected regiments from Paris and Marseilles, and the civil population were suffering from disorder, want, and misery of every kind, the disease did not spread.

The immunity of Lyons is now a generally recognized fact in France, and the city derives a considerable profit from it; for the rich people of Paris and Marseilles, whose circumstances permit it, are accustomed to flock to Lyons like sheep as soon as cholera breaks out in their homes, and readily pay a good price for the patient hospitality of the people. Formerly, if one asked in Lyons why the city was so happily and so strikingly spared, he would not be referred to the unusual cleanliness and comfortable life of the common people, nor to the splendid drinking-water, for, before filtered Rhone water was introduced in 1859, this was very bad, but to the air, whose circulation through the confluent valleys of the Rhone and the Saone was so lively that it was always master over the imported cholera-poison, and would not let it develop. But if we compare the velocity of the wind as observed at the Lyons meteorological station with that of other places much afflicted with cholera, we shall not find the slightest difference in favor of Lyons. The plain of Languedoc, over which the *mistral* blows so often, unroofing houses, uprooting trees, and destroying ships in the very harbor of Marseilles, is not seldom visited by epidemic cholera. Later investigations show that nothing is left with which to explain the immunity of Lyons but the condition of its soil. Apart from the size of the city, this immunity is not more striking—it is, in fact, not so striking—than that, for example, of Versailles, where, notwithstanding a constant daily and hourly communication with Paris, cholera has never broken out in an epidemic form. Decaisne has shown that the condition of the soil only can be regarded as bearing upon the immunity of this place.

Analogous facts may be found wherever the spread of cholera or typhoid is earnestly investigated. The beautiful city of Salzburg, which is now so hospitably entertaining the Association of Naturalists and Physicians, belongs to the number of fortunate cities that have so far been spared cholera-epidemics, notwithstanding numerous refugees from cholera have collected here when the disease prevailed in Austria and Southern Bavaria; among whom cases have occurred without the infection passing over to the city. Only in the winter of 1873–'74, when a severe outbreak of cholera occurred in the prison establishment at Laufen, did weak signs appear in Salzburg, showing that at least certain quarters of the city were not absolutely and invariably protected against cholera. So Lyons was made aware once, in

1854, that the whole city was not insusceptible to it. The Lyonnese were not willing to acknowledge this, for they had boasted too much of their immunity; but they asked, What do a few hundred cases of cholera in fifty years amount to in comparison with the total population (400,000 souls) of the city? We should not treat the subject in this way, but should rather ask, How many inhabitants has the part of the city which, even if it was only once, had a considerable number of cases of cholera? and then the reply can not be evaded that the suburb of Guillotiere suffered from a decided cholera-epidemic in 1854. This once-occurring epidemic was associated with an equally rare abnormal drought and a long-continued low stage of water in the Rhone, such as had not been observed since 1826. So Salzburg might at some time be visited with cholera, at least here and there, if the sky should obstinately keep its gates closed for an unusually long time, and the disease were in its neighborhood.

The reports of the cholera commission for the German Empire contain a large number of proofs of the influence of the locality, particularly of the soil, among which I might especially mention the reports of Gunther on the spread of cholera in the kingdom of Saxony, and of Pistor on the government district of Oppeln. Both these investigators, not satisfied with considering only the last visitation of cholera in 1872–'74, have also included within the circle of their inquiry all the outbreaks that have come to knowledge since the appearance of the disease in Europe in 1832. The confirmation of the localistic theory of cholera and other epidemics can no longer be put in question; and, if it were the only result reached by the German cholera commission, the money appropriated to the investigation would have been well spent.

We may now ask, What can there be in the soil that can exert so powerful an operation for good or evil upon our health? To this question, so far as concerns injury to health, the answer may be returned that, in all probability, the property is derived from the minute organisms or their products, of which many million individuals can be put within the area of the head of a pin, and which inhabit the porous soil from the surface down to a great depth—organisms which are capable of being injurious or harmless, or even useful to us, as we are already acquainted with injurious and harmless and useful plants and animals. They have heretofore been invisible to us, having only just been brought to knowledge, in the course of recent investigations in vegetable and animal physiology and pathology, by means of the microscope and experimental cultivation. A prominent vegetable physiologist, Naegelli, has accurately and clearly described them with direct reference to their hygienic significance in his well-known work, "Die niederen Pilze in ihren Beziehungen zu den Infectionskrankheiten und der Gesundheitspflege." Their mysterious presence recalls the ancient belief in invisible spirits that were accustomed to rise out of the earth, float in the water, and cast gloom over the spots haunted by them. Naegelli called a soil that produces or favors epidemics a disease-bearing (*Siechhaft*), and its opposite a healthful (*Siechfrei*). We must not conclude that only a locality of the former kind harbors molds and similar lower organisms, and one of the latter kind does not, or that these organisms reach us only from the former and not from the latter; on the contrary, they are always present everywhere. If they sometimes appear deleterious, at other times harmless, the conviction is forced upon us, either that the same species do not occur universally, or that the same species assume different properties at different places, under different circumstances, and at different times; that is, that they are only here and there and occasionally poisonous. If either is the case, the medium in which they live must exercise a very great influence upon them; and, so far as this medium is the soil, we have to investigate the conditions which it offers for the growth of these organisms and the communication of them to men. It must be admitted that mycology has so far given us very little light on this point, and many problems respecting it are still waiting to be solved; but it is already well established that hygiene as well as agriculture has much to do with the ground.

Some advance had already been made in the investigation of the hygienic relations of the soil before molds were mentioned as causes of infectious diseases. The simple observation that such diseases occurred or did not occur under certain conditions, of the soil was enough to provoke this. It had already become possible, without knowing the more immediate causes, to make an unhealthy soil healthy. The best known examples of this kind are given in the cases of intermittent fevers and malarial soils, in which the deleterious properties have been wholly or partly remedied by drainage and the drying up of the subsoil, and the fertilization and cultivation of the surface. Tommasi-malaria of Rome and the former drainage of the Roman hills, to the effect that the ancient Romans suffered much less from fevers than the Romans of after-times and of to-day. The archæologist De Tucci having called attention to some underground canals of a peculiar kind, called *cuniculi*, in the Roman hills, Tommasi examined them, and found that they were designed exclusively to drain the hills, and that they were now choked up and inoperative. Formerly, he thinks, they were so familiar that the ancient Roman writers did not think it worth while to speak of them; they passed into forgetfulness during the eruptions of the barbarians and the middle ages, and have now had to be discovered anew.

Measures directed against other infectious diseases that depend on the soil have not been without results, although the specific causes of the diseases are not known.

What are the conditions of soil favorable to epidemics?

It is an old experience that certain infectious diseases have their favorite seats in the so-called alluvial soils, in land subject to overflow. Alluvial soil consists chemically and geognostically of substantially the same mineral matters as the compact mountain-masses, from the disintegration of which it has originated—except that its physical aggregation is essentially different; and it is distinguished from rock soils by the great permeability for air and moisture arising from its great porosity, that is, from the spaces in which air and water, as well as organic matters, can find place. There are also kinds of rock which are very porous, and their behavior is not materially different from that of alluvial soils, as is shown by the cholera-epidemics in the Island of Malta.

In common life we can hardly conceive the extent of the porosity of the soil on which we dwell. Heavy, towering buildings often stand on a soil which is filled to the extent of a third of its volume with air. The investigation of ground-air has just begun, but it has already surprised us with some unexpected revelations. Ground-air is distinguished from the air that passes over the surface by the higher proportion of carbonic acid it contains, which increases, as a rule, with the distance from the surface, and to which our springs owe their charges of that gas. The carbonic acid is chiefly derived from organic matters and organic life in the ground, with which it increases and diminishes. Air brought by Zittel from the dead dry soil of the Libyan Desert, sealed in glass tubes, showed no larger proportion of carbonic acid than the free superficial air, but the ground-air from a palm-garden in the oasis of Farafreh yielded much carbonic acid. That this gas is mostly derived from organic changes is shown from the investigations of Fleck, Fodor, Wolffhügel, Möller, Wallny, and others, who found that the proportion of oxygen in ground-air was lower, while that of carbonic acid was higher, than in free air.

That the air in the soil does not become stagnant, but is always in motion, though sluggish, not only follows from physical laws, but may be easily proved by experiments and observations. Our houses are aired or ventilated in no small degree by the ground-air. Renk has been inquiring, with the aid of Recknagel's differential manometer, whether the air flows from the ground into the house or from the house into the ground, and has found that through most of the year the draft is from the ground into the house. He has also found that the ground-air, which is sucked into the house, brings dust with it, and other observers have shown that the same air also carries germs susceptible of development in suitable solutions.

It is thus easy to see how the soil affects our health without our having to eat it; the ground-air plays the part of an always ready intermediate agent, so far as concerns the molds. In this light it is easily seen why some houses sometimes have to suffer so badly from certain conditions of the soil, especially when they are badly ventilated. The movement of the air in a close house is many thousand times less active than where the circulation is free; and the air entering into the house suffers correspondingly less dilution than that passing into the free atmosphere, and leaves in it much more of what it brings up from the ground. While the house is heated during the cold season, and at night in the summer, while the air within-doors is warmer than the surrounding out-door air, the houses act as draught-flues, and suck air out from the ground as if they were cupping-glasses set over it. Experience has long taught us that it is most dangerous to sleep—that is, to pass the night—in such noted fever regions as the Pontine Marshes.

Many persons believe that the ground-air is an object whose existence is still pre-eminently theoretical, and that its practical influence is exceedingly remote. It does not present itself in this light to the physician who has had to deal with it. I am reminded of what the chief staff physician, Dr. Port, has remarked, as if by intuition, on the etiology of abdominal typhus, with immediate reference to military hygiene, and its bearings on the construction of barracks and camps. He says: "If we consider the danger to which the inhabitants of a disease-bearing soil are exposed by leaving their houses without protection from the soil, I might say by putting them on the ground barefooted, and, if we reflect that our most imposing palaces labor under this partial nakedness, we must of necessity receive the impression that there is some lack in our civilization. We have in this respect not only not excelled the most primitive constructions of the childhood of the building art, but have fallen behind them in a very important matter. We have no reason, from the hygienic point of view, to look down disparagingly on the pile-dwellings of some foreign races and the mud huts which our peasants still live in here and there: both of these classes of people, although in very different ways, have respected, in building, a hygienic principle that has escaped our architects. They have made their dwelling-places independent of the ground, in the former case by putting under them a grating of piles admitting a circulation of air; in the latter case by isolating the hut by means of a plaster floor. The superiority of these primitive dwelling-houses over our modern buildings can not be made to appear more clearly by any other example than by the sketch which Dr. Hirsch has given of an outbreak of cholera on the estate of Herr von Winter, chief health-officer of Dantzic:

"Nine houses stood in a group in front of the manor-house of the estate, and were inhabited by the farm-servants; seven of them had been rebuilt in timber with brick fillings, and furnished with cellars, which were perfectly dry; their ground-floors were lined with deal, were dry, airy, and kept clean; the manure-heaps were arranged in the manner that is common in rural districts. Two of the houses in the group had not been rebuilt; they were old mud huts, with low stories, without cellars; the rooms were not boarded up, but only plastered; and their condition seemed on the whole much more unfavorable than that of the others, while the manner of living of their inhabitants in other respects was in no way different from that of their neighbors in the modern cottages. About one hundred and fifty persons lived in all the nine houses. A woman, suffering from an attack of cholera, was taken into one of the new houses; three days afterward the first cases of sickness appeared in the neighborhood of this house, and the disease quickly spread to all the houses except the two old huts. The inmates of these houses had the same intercourse with their fellow-dwellers on the premises as the latter with each other; yet, while seventeen persons (or fifteen per cent. of the whole number) in the seven new houses were prostrated, not a case of sickness occurred in the old huts. The exemption of the latter was attributed to their being isolated from the ground by means of their plaster floors. The change of the other cottages into modern dwellings, with exposed foundations, was hygienically a reformation for the worse. We frequently commit the mistake in carrying out our ideas concerning the salubrity of a house, of confounding hygienic considerations with those of comfort."

Dr. Port brings forward other facts speaking for the influence of ground-air, and summarizes his view in the remark that he regards "a proper treatment of the soil as the first hygienic consideration, the chief prophylactic measure against certain infectious diseases, as the means by which we may make houses, barracks, tents, etc., dwelling-places free from disease. . . . From such dwellings we need not flee on the appearance of epidemics, but in them can bid defiance, as from a fortification, against disease; of such a dwelling we may say with truth, 'My house is my castle.'" It is very much to be desired that the building art could be turned, at least experimentally, in the direction indicated by Dr. Port. Practical hygiene is a little capable of being advanced without experiments as any other art; and where individuals can not experiment, the state should step in in the interest of the public weal, and provide the means for answering important questions.

